# A Developmental Switch in Neuronal Differentiation

**DOI:** 10.1371/journal.pbio.0030180

**Published:** 2005-04-26

Building an embryo is like building a house: everything has to be done at the right time and the right place if the plans are to be translated faithfully. On the building site, if the roofer comes along before the bricklayer has finished, the result may be a bungalow instead of a two-story residence. In the embryo, if the neurons, for example, start to make connections prematurely, the resultant animal may lack feeling in its skin.

On the building site, the project manager passes messages to the subcontractors, and they tell the laborers what to do and where. In the embryo, the expression of specific transcription factors (molecules that tell the cell which DNA sequences to convert into proteins) at different stages of development and in different places controls the orderly construction of the body.

Silvia Arber and her colleagues are studying the protracted process of neuronal differentiation in mice. Early in development, neurons are generated from dividing progenitor cells. Cell division stops soon after, and long extensions called axons grow out of the neurons in specific directions. When these axons reach their targets—peripheral tissues like the skin at one end, in the case of sensory neurons, and the spinal cord at the other—they form characteristic terminal branches. Finally, the nerves form contacts with other neurons so that they can pass messages on to the brain.

Many aspects of neuronal character are acquired through the expression of transcription factors in the progenitor cells or immediately after cell division stops. But Arber and her colleagues have been investigating whether an even later wave of transcription programs is needed for neuronal differentiation and circuit assembly in the sensory neurons of the dorsal root ganglia (DRG), structures containing the cell bodies of the sensory neurons. Previous work indicates that the release of molecules called neurotrophic factors by the neuron's target tissues directs the late expression of Er81 and Pea3. These ETS transcription factors (so called because they contain a region known as the erythroblast-transformation-specific domain) control late aspects of the differentiation of DRG neurons. What would happen, the researchers asked, if ETS proteins were expressed earlier? Would precocious ETS expression in DRG neurons also direct the appropriate neuronal developmental programs?

Arber's team made genetically engineered mice in which ETS signaling occurred either at the correct time or earlier, and examined the development of the proprioceptive sensory neurons, which are involved in the coordination of body balance. In vivo, they found that early initiation of ETS signaling disrupts the axonal growth of the DRG neurons, both to their peripheral targets and into the spinal cord, and perturbs the acquisition of terminal differentiation markers. In vitro, premature ETS signaling allows the DRG neurons to survive and grow in the absence of the neurotrophins normally required for these processes.

Arber and her coworkers conclude that the late onset of expression of ETS transcription factors induced by target-derived signals is essential for many of the later aspects of neuronal differentiation and circuit formation. During their differentiation, the researchers suggest, DRG neurons undergo a temporal switch in their ability to respond to ETS signaling. Further analysis of the mechanisms by which responses to transcription factor programs are altered over time during development will advance our understanding not only of neuronal differentiation but of other aspects of embryogenesis.

